# On-Chip Micro Mixer Driven by Elastic Wall with Virtual Actuator

**DOI:** 10.3390/mi12020217

**Published:** 2021-02-21

**Authors:** Toshio Takayama, Makoto Kaneko, Chia-Hung Dylan Tsai

**Affiliations:** 1 Department of Mechanical Engineering, Tokyo Institute of Technology, Tokyo 152-8552, Japan; 2 Graduate School of Science and Engineering, Meijo University, Nagoya 468-8502, Japan; mkaneko@meijo-u.ac.jp; 3 Department of Mechanical Engineering, National Yang Ming Chiao Tung University, Hsinchu 30010, Taiwan; dylantsai@nctu.edu.tw

**Keywords:** on-chip mixer, density control, PDMS, vibration

## Abstract

In this paper, we propose an on-chip micromixer driven by an elastic wall with a virtual actuator. The on-chip micro mixer is composed of a circular chamber surrounded by a ring-shaped channel under isolation with an elastic wall. When vibrational pressure is put on the driving channel by an actuator, the volume of the circular chamber changes through the deformation of the elastic wall, as if there exists a virtual actuator near the wall. As a result, the liquid in the circular chamber is pushed out and pulled through the neck channel. This action creates a swirling flow in the circular chamber while maintaining isolation from the driving channel. Through experiments, we confirmed the swirling flow under an isolated environment using an air-based valve. The advantage of this approach is that the micromixer can be designed with a single layer having a simple mechanism.

## 1. Introduction

Large vessels such as dishes with diameters of several centimeters are often utilized in cell culture experiments as well as chemical experiments. Such experiments generally require not only large spaces but also generate a considerable amount of wasted samples. To overcome these issues, microfluidic devices for chemical reactors or on-chip cell cultures have become popular in the last few decades [1,2,3,4,5,6], especially with different densities in a microchannel to observe the different reactions of the cells for nourishments or poisons of different densities. There are two methods for fabricating different-density areas in a microchannel. One is to create a gradient of solute in a large chamber by diffusion, and the other is to make several chambers with different densities. As for the former, Frevert et al. injected a small amount of high-density solution from a small nozzle into a large chamber, and the injected solution diffuses naturally to create a density gradient inside the chamber [7]. Keenan et al. arranged many small nozzles to create a rectangle-shaped gradient area [8]. Forry et al. succeeded in creating a gradient of CO${}_{2}$ gas density by using the permeability of dimethylpolysiloxane (PDMS) [9]. As for the latter, Meng et al. fabricated arrayed chambers of different densities along a channel [10].

An issue with fabricating chambers having different densities is that it takes considerable time to uniformly mix the solution in each chamber, and it may cause different reactions in the chamber. Therefore, a technique to mix the liquid inside a chamber is required to quickly achieve uniform density. However, owing to the low Reynolds number in microfluidic channels, realizing such a swirling flow in a microchannel is not easy. Vortices are effective for mixing liquids. There have been several studies that have researched the generation of vortices in microchannels. Common approaches for generating vortices in microchannels are to fabricate channels with edges or obstacles [11,12,13,14,15,16,17,18,19]. While these techniques are used for mixing laminar flow, they are not suitable for mixing stationary solutions in a chamber.

**Figure 1:**
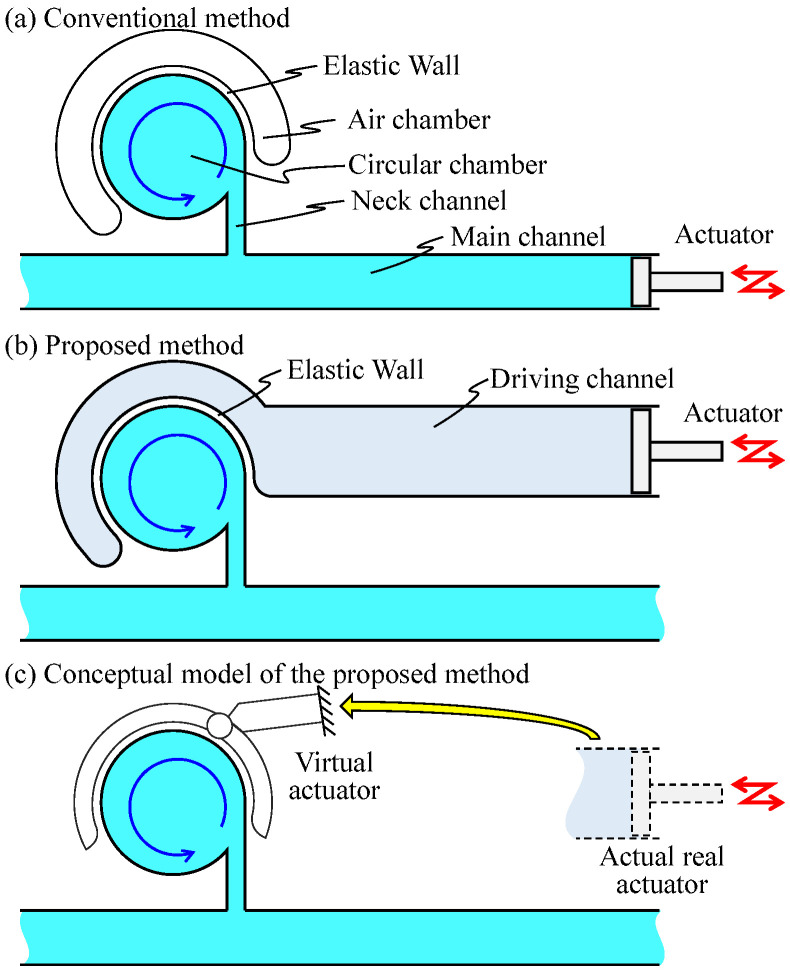
On-chip micro mixers. (**a**) Conventional method where the vibrational pressure is applied to the main channel. (**b**) Proposed method where the vibrational pressure is applied to the driving channel. (**c**) Image of the virtual actuator, a conceptual model of the proposed method; although the actual actuator is not present near the chamber, it behaves as if it is.

There are two methods to generate vortices in a chamber. One is to directly apply push/pull motion to the liquid by an actuator connected at the end of the main channel. For example, the actuator imparts reciprocated motions for the liquid in the main channel. Chung et al. enhanced the mixing efficiency in a chamber [20]. Glasgow et al. and Niu et al. enhanced laminar flow mixing by applying a periodic perturbation directly to the injected liquid via the main channel and the branched channel, respectively [21,22]. We have proposed Vibration-base Virtual Vortex Gear (V${}^{3}$G) wherein the vibrational pressure is applied to the fluid in the main channel and generates a swirling flow inside the circular chamber attached to the side of the main channel via a narrow neck channel by using a partial energy applied to the main channel, as shown in Figure 1a. The air chamber is used to enhance the side wall deformation of the circular chamber [23]. The other is to apply the force indirectly to the liquid to be mixed. Oberti et al. applied a vibrational force to the entire microchip to enhance laminar flow mixing [24]. Ahmed et al. used trapped air bubble/bubbles in the microfluidic channel to excite the vibration motion applied to the entire microchip to generate vortices [25,26]. Hayakawa et al. made small pillars inside the microfluidic channel to excite the vibration motion applied to the entire microchip to control the vortex flow [27]. Shang et al. attached piezoactuators on a chip to deform its top surface to apply the force to the liquid inside the chip [28]. Hansen et al. designed a PDMS chip with multiple layers and applied pressure in a ring-shaped chamber through the channel of the other layer [29].

In this paper, we propose an on-chip micromixer driven by an elastic wall, as shown in Figure 1b where the elastic wall is driven by the actuator attached at the end of the driving channel. When the vibrational pressure is put on the driving channel by the actuator, the volume of the circular chamber changes through the deformation of the wall, as if an actuator is surrounding the circular chamber and clamping it. Considering that the actual actuator does not exist near the chamber, even though it behaves as if it does, we call it a virtual actuator, as illustrated in Figure 1c. As a result, the liquid in the circular chamber is pushed out and pulled through the neck channel. This action creates a swirling flow in the circular chamber. Owing to certain circumstances, the mixing needs to be isolated from other parts of the microfluidic system. If air is injected into the main channel, it functions just as a valve, and the proposed on-chip micromixer can mix the liquid inside it, while keeping it perfectly isolated from other liquids. There are advantages for the proposed method, such as powerful swirling flow, perfect isolation between the driving liquid and chemical solution, simple single-layer design of PDMS, and an air-based valve to maintain constant density.

The remainder of this paper is organized as follows. In Section 2, we explain the concept and working principle of the on-chip micromixer driven by an elastic wall with a virtual actuator in detail. In Section 3, we explain the experimental setup and procedures. In Section 4, we show the experimental results. In Section 5, we provide discussions. In Section 6, we provide concluding statements and future plan.

## 2. Basic Concept and Working Principle

Figure 1a shows a typical example of an on-chip micromixer with an elastic wall driven by an actuator implemented at the end of main channel, where the air chamber with an elastic wall enhances the vibration in the circular chamber. Suppose that the actuator is oscillating in the main channel. During this action, a part of the chemical liquid will push in and pull back from the circular chamber. As the center line of the neck channel is shifted from the center line of the chamber, a counter-clockwise flow will occur, as shown in Figure 1a However, fluid energy is lost when it passes through the slender neck channel owing to the flow resistance. As a result, the flow force pushing the elastic wall becomes weak, thereby eventually reducing the swirling velocity. As the actuator head makes contact with the chemical liquid directly, this causes contamination in the channel. Contamination should be avoided in cell culturing, drag manufacturing, and chemical mixing.

**Figure 2:**
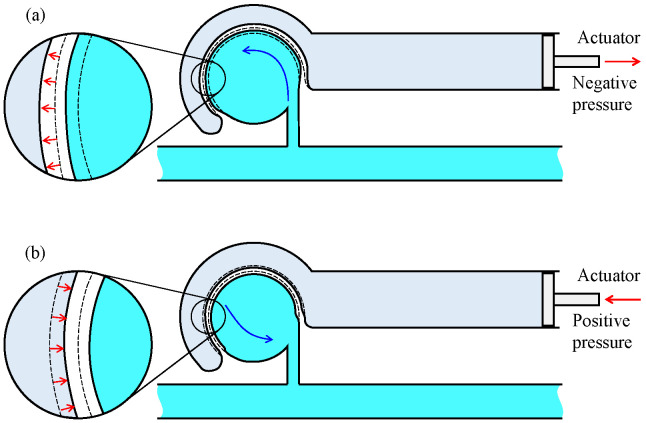
Principle of generating swirling flow inside the circular chamber. (**a**) Deformation of wall when a negative pressure is applied. (**b**) Deformation of wall when a positive pressure is applied.

To address these issues, we propose an on-chip mixer driven by an elastic wall with a virtual actuator, as shown in Figure 1b, where two isolated channels are implemented. One is the driving channel, where a real actuator is connected at the end of the channel, and the other is the main channel where a circular chamber is connected. Suppose that the driving and the main channels are filled with water and a particular liquid, respectively. When the vibrational pressure is put on the driving channel by a real actuator, the volume of the circular chamber changes through the deformation of the elastic wall, as if there exists an actuator near the wall virtually, as shown in Figure 1c. As a result, the liquid in the circular chamber is pushed out and pulled through the neck channel. This action creates a swirling flow in the circular chamber while maintaining isolation from the driving channel. The energy of the pushed water is transmitted to generate a powerful swirling motion through the deformation of the elastic wall. Owing to the mechanical configuration with two isolated channels, we are completely released from contamination issue owing to the mixing of two liquids. Figure 2 shows the principle of the deformation of the elastic wall depending upon the vibration phase of the real actuator. When negative pressure is applied to the driving channel, the liquid existing in the neck channel flows into the circular chamber along the wall because of its inertia, as shown in Figure 2a. On the contrary, when a positive pressure is applied, the liquid in the circular chamber is uniformly pushed out to the neck channel, as shown in Figure 2b. Owing to the difference in these flow patterns, an anti-clockwise torque is generated in one cycle of push/pull motion, and by repeating this motion continuously, a large swirling flow is generated. The proposed on-chip mixer is designed by a single layer with a simple design.

## 3. Experimental Setup and Procedures

**Figure 3:**
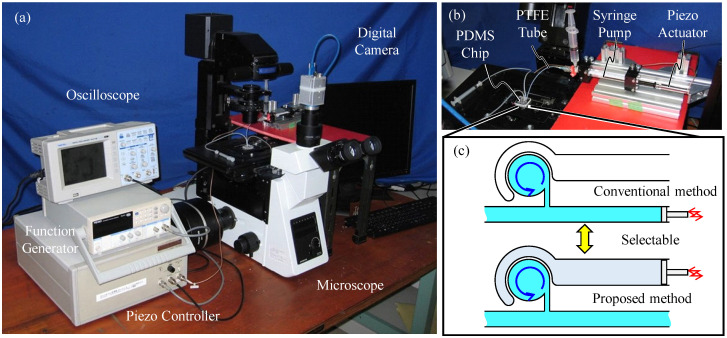
Experimental setup. (**a**) Overview of the microscope. (**b**) Dimethylpolysiloxane (PDMS) chip and actuator. (**c**) Schematic of the connection between the channel and the actuator.

Figure 3 shows the experimental setup where (a), (b), and (c) are the overview of the system including the microscope, PDMS chip, and actuator, and the schematic of the PDMS chip. The PDMS chip is the microfluidic channel made by PDMS bonded on a slide glass. The development method and actual design of the PDMS chip are introduced in the next paragraph. A function generator (TEXIO, FG-274, Yokohama, Japan) generates a square wave and the piezo controller (MESS-TEK, M26109B) amplifies the square wave 15 times. It then sends the signal to the piezo actuator (MESS-TEK, PSt 150/5/40 VS10, Wako, Japan) to drive the vibrational motion to the channel surrounding the circular chamber. The oscilloscope is used for confirming the actually applied voltage to the piezoactuator. The energy consumption of the piezo actuator is generally low. The energy that is stored in the piezo actuator during one stroke motion is $CV^{2}/2$, where C and V are the capacitance of the piezo actuator and the applied voltage, respectively. Therefore, if Joule heat is neglected, we can estimate the energy consumption using $E=HCV^{2}/2$. Here, E and H are the wattage and the driving frequency, respectively. The capacitance, C, of the piezo actuator used is 1600 nF; therefore, E is effectively limited. In the test, the voltage and frequency applied by the function generator are 4 V and 1000 Hz, respectively. We can assume the energy consumption to be 2.88 W, based on the equation and the amplification of the piezo controller of 15 times. The electrical devices in Figure 3 account for a large footprint. This is because the devices are intended for multi-purpose use. If the electric circuit is developed solely for this actuator, the electrical devices would be smaller, and consequently, the footprint of the system would be reduced. A syringe pump is connected to the PDMS chip via a PTFE tube and actuated by a piezoactuator to apply vibrational pressure to the elastic wall of the circular chamber. To compare the differences in mixing motions of the conventional method and the proposed method, we designed the channel such that it can be used for two experiments only by changing the connection of the syringe pump to the PDMS chip, as shown in Figure 3c. To evaluate the mixing motion and compile the mixing ability of the solutions and small particles, we used two types of liquid for visualization, where one was colored water and the other was water with microbeads. For the colored water, we mix red food coloring with pure water until saturation of concentration. For the colored water, we performed the experiment five times. For water with microbeads, the microbeads (Estapor Microspheres Latex Calibrated Particles Ref: K100) were mixed with pure water at 5 wt%. As the microbeads easily adhere inside the microchannel, performing experiments repeatedly is difficult. Therefore, the experiment for the microbeads is conducted only one time for each method. The image of the microscope was captured by using a digital high-speed camera (Shodensha, CHU130EX, Osaka, Japan) and its luminance values are used for data analysis.

**Figure 4:**
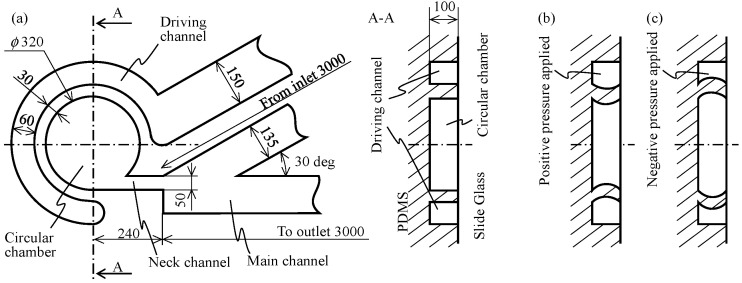
Design specification of the proposed channel. (**a**) Dimensions of both frontal and cross-sectional views of the circular chamber. (**b**) Deformation of elastic wall in cross-sectional view when positive pressure is applied to the driving channel. (**c**) Deformation when negative pressure is applied to the driving channel.

Figure 4a shows the design specification of the channel for both frontal and cross-sectional views, where the height of all channels and width of the elastic wall are 100 μm and 30 μm, respectively. An inlet port and an outlet port are present at the end of the main channel, to insert a tube for injecting the liquid to be mixed. The distance between the neck channel and these ports is also shown in the figure. The mold of the microfluidic channel is prepared using SU-8 via the usual photolithographic technique, and PDMS in the liquid state is poured over the mold and cured. The cured PDMS is then removed from the mold. Subsequently, it is bonded with the slide glass to form a PDMS chip containing the microfluidic channel. Figure 4b,c show the deformation of the elastic wall in the cross-sectional view between the circular chamber and the driving channel, where (b) and (c) show two cases of positive and negative pressures applied to the driving channel, respectively. The actual deformation is not very large, and Figure 4b,c are drawn as exaggerated. If the width of the wall decreases, the deformation would increase, leading to a more powerful swirling flow and more effective mixing. The required wall thickness is based on the aspect ratio of the wall height and thickness. If the aspect ratio of the wall is high, it becomes difficult to remove the cured PDMS from the mold without breaking the wall structure. Thus, we adopted an aspect ratio of 100:30, to ensure that both effective mixing and consistent fabrication are achieved. The resonance frequency depends on the design of PDMS chip and the experimental setup. We first examined the resonance frequency by applying an appropriate voltage and observed that it is 1000 Hz with a 4 V input. The following experiments are done by using these frequency and voltage values.

Experimental plans are based on the following procedures, where experiments 1, 2, and 3 are by utilizing the conventional method as shown in Figure 1a, by utilizing the proposed method as shown in Figure 1b, and by using the proposed method with the air-based valve that injects air into the main channel to isolate the liquid inside the circular chamber from other liquids, respectively.

**Figure 5:**
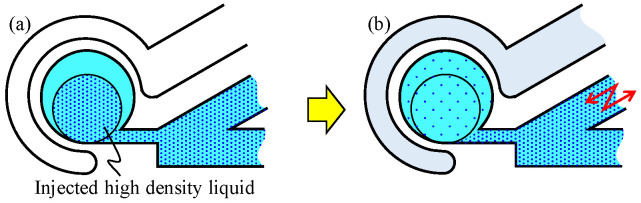
Procedure for experiment 1. (**a**) Initial state. (**b**) Applied vibrational pressure force on main channel.

Experiment 1 (Conventional Method): At first, all channels except for the driving channel are filled with pure water. Subsequently, the colored water or water containing microbeads is injected into the main channel. By manually controlling the flow speed, we injected the high-density liquid into the circular chamber in a VVG manner [30] until it occupies a predetermined circle whose area is half of the circular chamber, as shown in Figure 5a. Note: To inject an arbitrary amount of the liquid into the circular chamber in the VVG manner, a highly accurate flow speed is not required because the sensitivity for the flow speed of VVG is not that high. Therefore, it can be easily injected manually. Next, we connect the syringe pump actuated by the piezoactuator to the main channel, Then, the vibration is applied to the main channel, as shown in Figure 5b.

**Figure 6:**
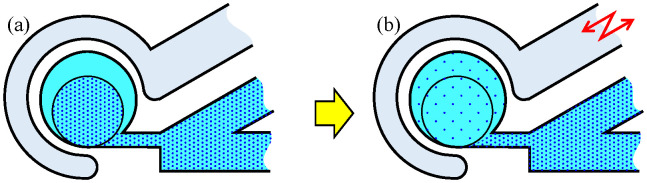
Procedure for experiment 2. (**a**) Initial state. (**b**) Applied vibrational pressure force on driving channel.

Experiment 2 (Proposed Method): At first, all channels are filled with pure water. Then, the colored water or the liquid containing microbeads is injected into the circular chamber in a manner similar to that used for the preparation in experiment 1, as shown in Figure 6a. Subsequently, the vibration force is applied to the driving channel, as shown in Figure 6b.

**Figure 7:**
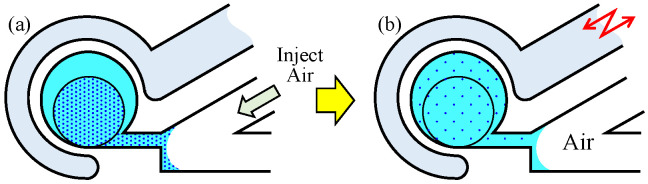
Procedure for experiment 3. (**a**) Initial state. (**b**) Applied vibrational pressure force on driving channel.

Experiment 3 (Proposed method with the air-based valve): At first, when taken in experiment 2, the colored water or liquid containing microbeads is injected into the circular chamber. Subsequently, a syringe with air is connected to the main channel to inject air into the main channel, as shown in Figure 7a, Then, the vibration is applied to the driving channel, as shown in Figure 7b.

**Figure 8:**
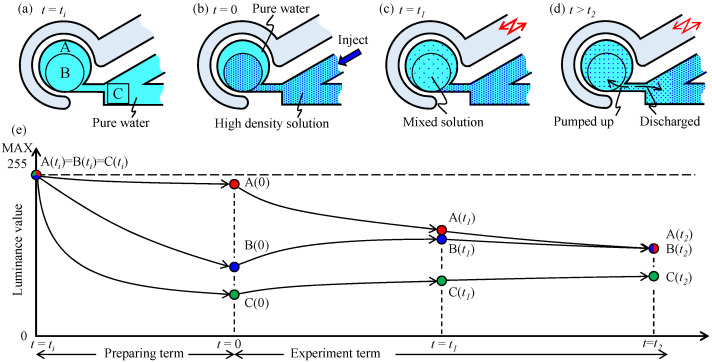
Expected relation between the luminance values in three regions and action in experiment 2. (**a**) The condition immediately after pure water is supplied. (**b**) Initial condition for the experiment. (**c**) After mixing inside the circular chamber. (**d**) After long term mixing. (**e**) Expected change in the luminance value of each region.

For example, we suppose experiment 2 where the colored water is utilized. Figure 8 visually explains the expected luminance value in various areas with respect to time, where regions A, B, and C shown in Figure 8a are windows for observing the luminance in each region, where the areas of regions A and B are the same, and region C is inside the main channel and fixed at the side of the neck channel. We evaluated the luminance value in these regions using ImageJ (https://imagej.nih.gov/ij/ (accessed on 20 February 2021)) with respect to time. The resolution of the luminance value is 256 degrees because of the color definition of the image. The luminance values in regions A, B, and C are defined by A(t), B(t), and C(t) at time t, respectively. Figure 8 illustrates the expected trends of luminance at different regions. Quantitative values of the experimental results will be shown in the next section. Figure 8a shows the condition immediately after pure water is supplied for all channels and the circular chamber at $t=t_{i}$. As the transparency of pure water is high, the luminance values in regions A, B, and C are similarly high, as shown in Figure 8e. To set up the initial condition for the experiment, we inject a solution with a high density from the main channel, as shown in Figure 8b. When we injected a high-density solution from the main channel, the solution flowed into regions B and C; subsequently, the solution diffuses between regions A and B. Owing to this behavior, we usually obtain the following order where A(0)>B(0)>C(0), as shown in Figure 8e. This means that at *t* = 0, C(0) is the highest density, B(0) is the second highest density, and A(0) is the lowest density, respectively. After the vibration pressure is applied to the driving channel at t>0, the liquid in regions A and B are mixed gradually, as shown in Figure 8c, where the order of the luminance value is $A\left(t_{1}\right)>B\left(t_{1}\right)>C\left(t_{1}\right)$. For the initial phase of t>0, A(t) gradually decreases, while B(t) gradually increases, as shown in Figure 8e. Simply speaking, the time for completing mixing is given by $t_{2}$ satisfying $|A\left(t_{2}\right)-B\left(t_{2}\right)|<\epsilon \left(\epsilon >0\right)$, where ϵ is small. While $|A\left(t_{2}\right)-B\left(t_{2}\right)|<\epsilon$ for $t>t_{2}$, there might be some options, such as the liquid inside the circular chamber gradually becomes a high-density one owing to the pumping up effect from the main channel, as shown in Figure 8d.

A naive issue in the experiment is that the amount of initially injected high-density liquid into the circular chamber is slightly different in each experiment. To suppress the effect of the difference in the quantity of high-density liquid among the experiments, we newly introduce the following equation so that we can normalize the data with respect to time.

(1) $$X_{n}\left(t\right)=\frac{X(t)-C(0)}{A(0)-C(0)},$$

where $X_{n}\left(t\right)$, and X(t) are the normalized luminance value of region X at time *t* and the measured luminance value of region X at time *t*, respectively, where *X* is either *A* or *B* or *C*. By using this normalized luminance value, we can compare the luminance values of all experiments using colored water. In experiment 3, we injected air into the main channel so that region C may be filled with air, which makes an air-based valve for stopping liquid mixing between the main channel and the circular chamber. Before we inject air, we measured the luminance value of region C and used it instead of C(0).

To evaluate the mixing degree between regions A and B, we define the mixing index $D_{A-B}\left(t\right)$ with the following equation.

(2) $$D_{A-B}\left(t\right)=A_{n}\left(t\right)-B_{n}\left(t\right)$$

The physical meaning of the mixing index is that when the luminance values are the same between regions A and B, $D_{A-B}\left(t\right)$ becomes zero. Simply speaking, if $D_{A-B}\left(t\right)$ is zero, the mixing action is completed in the circular chamber.

To evaluate the non-mixing degree between regions A + B and C, we define the non-mixing index $D_{AB-C}\left(t\right)$ using the following equation.

(3) $$D_{AB-C}\left(t\right)=\frac{A_{n}\left(t\right)+B_{n}\left(t\right)}{2}-C_{n}\left(t\right)$$

The physical meaning of the non-mixing index is that the liquids between regions A + B and C do not interact each other under a high $D_{AB-C}\left(t\right)$, while they interact significantly under $D_{AB-C}\left(t\right)=0$. From the viewpoint of flow blocking at the neck channel, a higher $D_{AB-C}\left(t\right)$ is better.

## 4. Experimental Results

**Figure 9:**
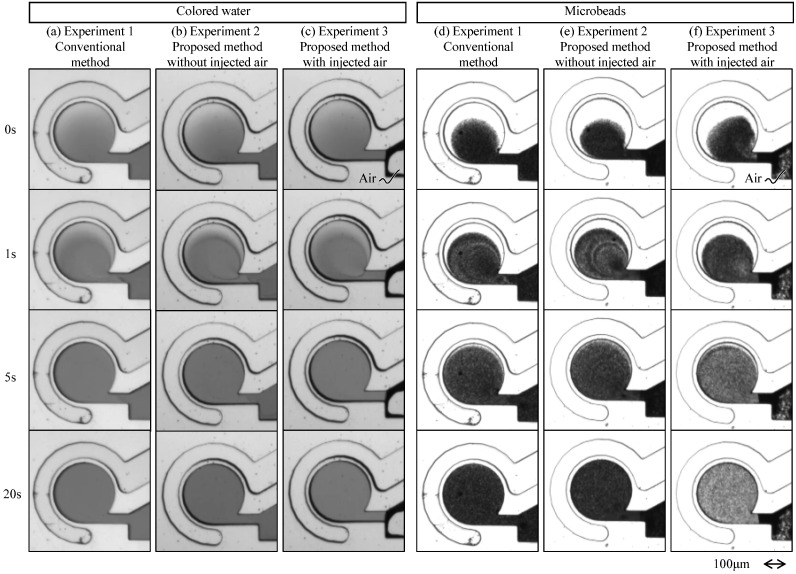
Visualized swirling flow in the circular chamber, where (**a**–**c**) are the results of experiments 1, 2, and 3 by using colored water, and (**d**–**f**) are those of using water with microbeads, respectively.

Figure 9a–f are the captured images of the experiments 1, 2, and 3, by using colored water, and those by using microbeads. Figure 9 shows that the luminance values gradually become uniform in the circular chamber with respect to time, after the vibration motions start.

**Figure 10:**
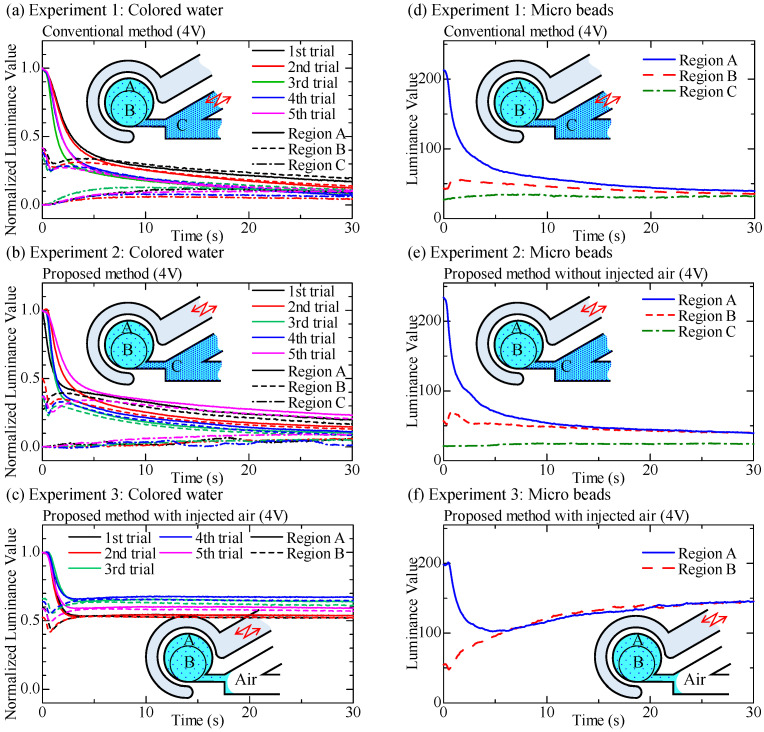
Experimental results of the mixing, where (**a**–**c**) denote experiments 1, 2, and 3 by using colored water, and (**d**–**f**) are those of using microbeads, respectively.

Figure 10 shows the normalized luminance for all experiments conducted by using colored water obtained by Equation (1) and the luminance value by using microbeads. The sampling rate is 0.05 s. While there are some variations, we can see a general tendency for $A_{n}\left(t\right)$,$B_{n}\left(t\right)$, and $C_{n}\left(t\right)$ with respect to time. As for the colored water, by the definition of Equation (1), $A_{n}\left(t\right)$ always starts with $A_{n}\left(0\right)=1.0$ and decreases gradually with respect to time and $C_{n}\left(t\right)$ always starts with $C_{n}\left(0\right)=0.0$ and gradually increases with respect to time. The high-density liquid is pumped from the main channel into the circular chamber. $B_{n}\left(t\right)$ is just between $A_{n}\left(t\right)$ and $C_{n}\left(t\right)$, except Figure 10c where air is injected into the main channel to block liquid mixing between the circular chamber and the main channel. These results by using colored water focusing on the luminance values of regions A and B are not the same perfectly; Nevertheless, they converge to the stable values as shown in Figure 10c. Figure 10c also shows that the luminance values of regions A and B do not decrease any more after they are mixed. As for the water with microbeads, the experimental results are shown in Figure 10d–f, where air is not injected into the main channel in Figure 10d,e, and air is injected in Figure 10f. Experimental results using microbeads show similar behaviors in $A_{n}\left(t\right)$, $B_{n}\left(t\right)$, and $C_{n}\left(t\right)$ in both the proposed and the conventional methods in experiments 1 and 2. However, when air is injected into the main channel for blocking liquid mixing between the circular chamber and the main channel (experiment 3), the behaviors of the luminance values of regions A and B are different from those in Figure 10d,e. After the luminance values of regions A and B become the same, the luminance values of both regions A and B increase gradually with respect to time, as shown in Figure 10f. The density in the chamber becomes low, and the microbeads in the chamber partially exit the circular chamber; Nevertheless, the circulation chamber is isolated by the air-based valve.

**Figure 11:**
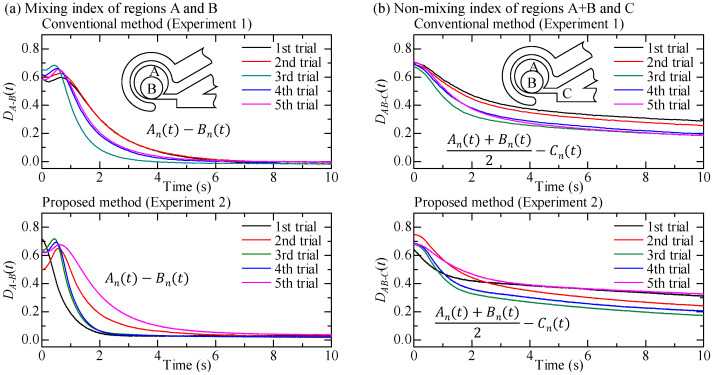
The mixing index and the non-mixing index of experiment 1 and experiment 2. (**a**) Difference in luminance values of regions A and B. (**b**) Difference in luminance values of the average of region A + B and region C.

Figure 11a shows the mixing index indicating the difference of luminance value between regions A and B. Figure 11a shows that the luminance value becomes the same for all experiments in less than 10 s in both the conventional and proposed methods. As a general tendency, completing the mixing action is faster in the proposed method than in the conventional method. Figure 11b shows the non-mixing index indicating how much the liquids move between region A + B and region C. Figure 11b shows that the luminance value becomes a non-negligible difference between region A + B and region C. In general, the luminance value of the proposed method is larger than that of the conventional method. In other words, after completing the mixing action, there is little liquid flowing between region A + B and region C in the proposed method, while more liquid flows in the conventional method.

## 5. Discussion

**Figure 12:**
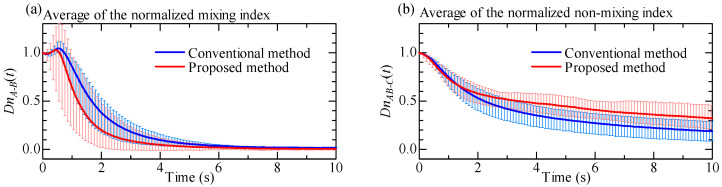
Averaged lines of the normalized mixing index and normalized non-mixing index. (**a**) Normalized mixing index between regions A and B. (**b**) Normalized non-mixing index between regions A + B and C.

To achieve statistical evaluation for both the mixing and non-mixing indices, First, we modify Equations (2) and (3) by the following Equations (4) and (5) so that all modified parameters can start from unity. For simplicity, we focus on the data obtained using colored water.

(4) $$Dn_{A-B}\left(t\right)=\frac{D_{A-B}\left(t\right)-D_{A-B}\left(30\right)}{D_{A-B}\left(0\right)-D_{A-B}\left(30\right)}$$

(5) $$Dn_{AB-C}\left(t\right)=\frac{D_{AB-C}\left(t\right)}{D_{AB-C}\left(0\right)},$$

where $Dn_{A-B}\left(t\right)$ and $Dn_{AB-C}\left(t\right)$ denote the normalized mixing index inside the circular chamber and the normalized non-mixing index between the circular chamber and the main channel, respectively. Figure 12 shows the behaviors of average value of these parameters with respect to time, where Figure 12a,b are the normalized mixing index and the normalized non-mixing index, respectively. From Figure 12, we can clearly observe that the normalized mixing index decreases more sharply under the proposed method than under the conventional method. As for the non-mixing index, the behaviors for both methods are similar up to 1.5 s, but there is a noticeable gap in the behaviors at *t* = 10 s.

To quantitatively compare the mixing speed, non-mixing speed, and gap, we independently perform numerical derivatives for each data. Table 1 shows the result where the (a) maximum decreasing value of the normalized mixing speed, (b) maximum decreasing value of normalized non-mixing speed, and (c) gap at *t* = 10 s, respectively. The values in Table 1 (a) and (b) are the time derivatives of the mixing index and the non-mixing index. They represent the slope of a graph. If the value is negative, the index is decreasing with respect to time, indicating that the mixing is progressing. The smaller the value, the higher is the mixing speed. By using these data, we executed the T-test. First, let us focus on the normalized mixing speed. Table 1 shows that the *p*-value is 0.0498, This indicates a significant difference in the normalized mixing speed between the proposed and conventional methods, and the proposed method is 1.69 times faster than the conventional method. Now, let us focus on the normalized non-mixing speed. As for the normalized non-mixing speed, we do not see any significant difference between the proposed and conventional methods. Finally, we focus on the gap at *t* = 10 s. Although there are no significant differences for both the gaps at *t* = 10 s, we can visually see a clear difference between the proposed and the conventional methods. From this analysis, we can statistically confirm that the proposed method can mix liquid even faster than the conventional method.

As for the phenomenon where the luminance value increases gradually when microbeads are used, as shown in Figure 10f, this phenomenon is discussed in Appendix A in detail.

## 6. Concluding Remarks

We proposed an on-chip micromixer driven by an elastic wall with a virtual actuator. The proposed method can mix the liquid in the circular chamber 1.69 times faster than that of our conventional method. As the driving channel is completely separated from the main channel, we can avoid mixing both the liquid in the driving channel and the liquid in the main channel. As a result, it can avoid contamination from the liquid in the driving channel. Moreover, the proposed microfluidic channel can be made using a single-layered PDMS chip, thereby contributing to manufacturing it with low cost and simple disposable configuration. Moreover, if the mixing liquid is a solution, mixing can happen with maintaining its density in the chamber by injecting air into the main channel to isolate the liquid in the chamber from other liquids. This option can be conveniently utilized depending upon the application. These advantages are suitable for chemical and biological experiments. In the future, we plan to arrange many chambers with different densities simultaneously. We did not observe the appearance of air bubbles during the experiments. If air bubbles appear during long term operation with the air-based valve, they are likely caused by mixing with air from the neck channel. In such cases, the bubbles can be removed by applying pressure to the main channel, because PDMS is a gas permeable material. In this regard, we need to confirm if air bubbles appear during long term operation and whether they can be eliminated. Moreover, the current volume of the proposed micromixer was determined based on experiments to ensure the convenient generation of a swirling flow. The inertia of the flow-in liquid is a key parameter governing the swirling motion, as shown in Figure 2a. Therefore, if the volume increases, less swirling in the chamber is expected. As a future work, we plan to address this limitation on the volume of the device to realize wide applicability.

## Figures and Tables

**Table 1:** Numerical evaluation of the mixing speed. (**a**) Decrease in mixing index speed between regions A and B. (**b**) Decrease speed of the non-mixing index between regions A + B and C. (**c**) Gap at *t* = 10 s.

	(a) Minimum Value of		(b) Minimum Value of		(c) ${\mathrm{Dn}}_{\mathrm{AB}-C}\left(10\right)$	
	${\mathrm{dDn}}_{A-B}\left(t\right)/\mathrm{dt}$		${\mathrm{dDn}}_{\mathrm{AB}-C}\left(t\right)/\mathrm{dt}$			
	**Conventional**	**Proposed**	**Conventional**	**Proposed**	**Conventional**	**Proposed**
1st trial	−0.4677	−1.1419	−0.2950	−0.3836	0.2632	0.4583
2nd trial	−0.4970	−1.1294	−0.2650	−0.3684	0.2866	0.3065
3rd trial	−1.0213	−1.5580	−0.4910	−0.5211	0.0870	0.2095
4th trial	−0.7526	−1.5083	−0.3607	−0.4667	0.1691	0.2453
5th trial	−0.7420	−0.5421	−0.3782	−0.2676	0.1304	0.3857
Average	−0.6961	−1.1760	−0.3580	−0.4015	0.1573	0.3211
*p*-value	0.0498		0.4786		0.0544	

**Figure A1:**
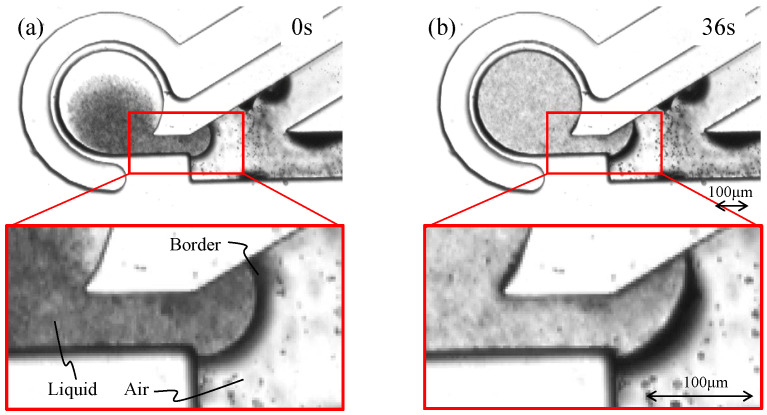
The growth of the dark area of the border between the water and air, where (**a**,**b**) are before driving and 30 s after driving, respectively.

**Figure A2:**
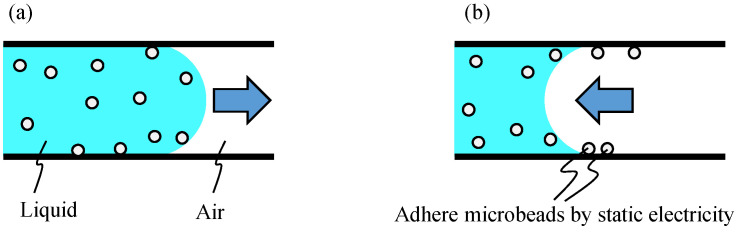
Mechanism to adhere microbeads at the border of the liquid and air. (**a**) When the liquid moves to the empty area. (**b**) When the liquid moves backward.

**Figure A3:**
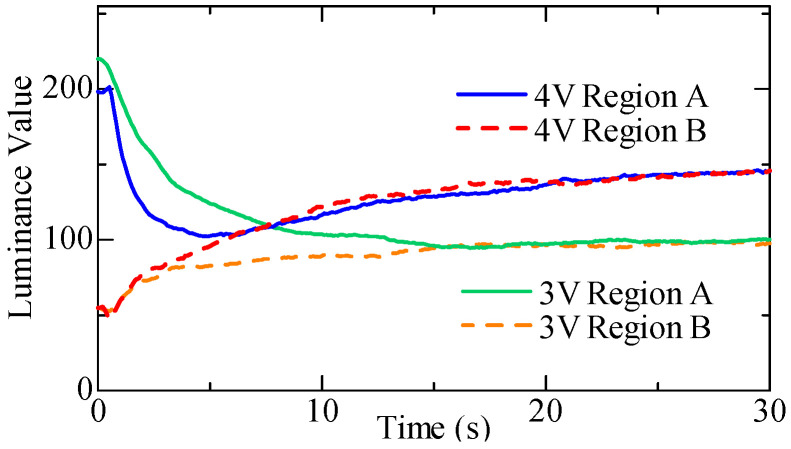
Experiment 3 using microbeads and applied voltages of 3 and 4 V.

## Data Availability

The datasets used and/or analyzed during the current study are available from the corresponding author on reasonable request.

## Appendix A. The Phenomenon of the Decrease of the Microbeads Density

In the experiment using microbeads, the luminance values of regions A and B become almost the same when the mixing is saturated, as shown in Figure 10f. An interesting behavior is that the luminance values of regions A and B increase after they become the same; nevertheless, the circular chamber is isolated by injecting air into the main channel. This phenomenon is caused by the microbeads. In the experiment, as shown in Figure 9, many microbeads are adhered inside the main channel. Therefore, unfortunately, we cannot observe the details near the neck channel. To confirm what is happening, we performed the same experiment using low-density liquid containing microbeads. Figure A1a and Figure A1b shows enlarged images of the border of the liquid and air before and after the vibrational force is applied for 36 s, respectively. In Figure A1a, the darkness of the border may be caused by optical refraction, and in Figure A1b the darkness area grew wide. We considered that the adhered microbeads enlarge the dark area.

A possible explanation is that the microbeads at the border of the liquid and air are shown in Figure A2. When the liquid moves in the right direction, the microbeads are carried by the flow, as shown in Figure A2a. When the liquid returns to the chamber, the microbeads are adhered to the wall by static electricity, as shown in Figure A2b. Once the microbeads are adhered to the wall, it is difficult to remove them even if the liquid returns in the next cycle of the motion. Thus, by repeating this phenomenon, the microbeads gradually become denser and the microbead density of the liquid in the circular chamber becomes low. We also examined the influence of the amplitude of the motion of the liquid by changing the applied voltage. Figure A3 shows the experimental results through which the increase in the luminance value is suppressed by reducing the applied voltage from 4 to 3 V.
